# Supplementary material for: Trends in malaria prevalence and risk factors associated with the disease in Nkongho-mbeng; a typical rural setting in the equatorial rainforest of the South West Region of Cameroon
Source: PLoS One. 2021 May 18;16(5):e0251380. doi: 10.1371/journal.pone.0251380 (PMC8130964; doi:10.1371/journal.pone.0251380)
Supplement: S1 File — (PDF) [file pone.0251380.s001.pdf]

QUESTIONNAIRE

Code........... **Date…………………**

The Burden of Malaria in Nkongho-Mbeng and Risk Factors Associated With the Disease

Section one: Demography

1. **Age**……………….
2. **Gender:** (1) Male ☐ (2) Female ☐
3. **Marital status:** (1) Married ☐ (2) Single ☐ (3) Widow ☐ (4) Widower
4. **Village:** (1) Dinte ☐ (2) Mbetta ☐ (3) Lekwe ☐
5. **Occupation:** (1) Famer ☐ (2) Teacher ☐ (3) Health worker ☐ (4) Business ☐

   (5) Others ☐ (specify……………………………….)
6. **Educational level:** (1) Never been to school ☐ (2) Primary school ☐ (3) Secondary school ☐ (4) High school ☐ (5) University and above ☐

Laboratory analysis

1). Microscopy: (1) positive ☐ (2) negative ☐

2). Parasite load…………………………

Section Two: Social Status

1. House type: (1) Mud block ☐ (2) Cement block ☐ (3) Others ☐ (specify…………)
2. Number of people in the household: ………………………..
3. Is there a ceiling?: (1) Yes ☐ (2) No ☐
4. If yes, type of ceiling: (1) Plywood ☐ (2) Zinc ☐ (3) Bamboo ☐ (4) others ☐

   (specify…………………………)
5. Do windows have mosquito nets?:(1) Yes ☐ (2) No ☐
6. Is there space at the gable ends of the wall: (1) Yes ☐ (2) No ☐

7. Type, type of toilet: (1) Pit latrine ☐ (2) Water system ☐ (3) Others ☐ (specify ……………………..)
8. Do you often stay out of your home late into the nights: (1) Yes ☐ (2) No ☐
9. What is your monthly income level: ………………………………

Section three: Malaria Management

1. **Have you ever had malaria:** (1) Yes ☐ (2) No ☐
2. **If yes, when did you last have malaria:** (1) This month ☐ (2) Five month ago ☐ (3) One year ago ☐
3. **(a) Where do you take treatment for malaria:** (1) Health center ☐ (2) Drug store ☐ (3)Herbalist ☐ (4) Automedication ☐ (5)Others ☐ (specify………………)

   (b) Which drug did you use: (1) Athermeter ☐ (2) Quinine ☐ (3) Don't know ☐

(4) Others ☐ (specify………………)

Section four: Vector control

1. Is there standing water within 20 meters around the house: (1) Yes ☐ (2) No ☐
2. Are there bushes around the house: (1) Yes ☐ (2) No ☐
3. Are there crops around the house: (1) Yes ☐ (2) No ☐
4. If yes, what type: (1) Plantain ☐ (2) Cocoa ☐ (3) Coffee ☐ (4) Cocoyam ☐ (5) Others ☐ (specify………………………..)
5. Do you have a mosquito net?: (1) Yes ☐ (2) No ☐
6. If yes, how frequent do you use it: (1) everyday ☐ (2)Some days ☐ (3) Don't use ☐
7. What is the state of the net: (1) Old ☐ (2) Fairly use ☐ (3) New ☐
8. Dose the net have holes: (1) Yes ☐ (2) No ☐
9. Have you ever seen a dead insect on your net:(1) Yes ☐ (2) No ☐

10. Have you ever seen a dead mosquito on your net: (1) Yes ☐ (2) No ☐

11. Where did you obtain the net: (1) Market ☐ (2) Health center ☐ (3) Campaign ☐

12. When did you obtain the net: (1) A month ago ☐ (2) 6 months ago ☐ (3) 1 year ago ☐ (4) Others ☐ (specify……………………….)

13. Do you use insecticide mosquito spray: (1) Yes ☐ (2) No ☐

14. If yes, how frequent do you use it: (1) everyday ☐ (2) Some days ☐ (3) Don't use ☐

15. Where do you carry your drinking water: (1) Spring ☐ (2) Stream ☐ (3) River ☐ (4) Others ☐ (specify………………………..)

16. How do you store water in the house: (1) Open containers ☐ (2) Close containers ☐ (3) Others ☐ (specify……………………….)

Thank you for your time
