# Supplementary material for: Trends in malaria prevalence and risk factors associated with the disease in Nkongho-mbeng; a typical rural setting in the equatorial rainforest of the South West Region of Cameroon
Source: PLoS One. 2021 May 18;16(5):e0251380. doi: 10.1371/journal.pone.0251380 (PMC8130964; doi:10.1371/journal.pone.0251380)
Supplement: Attachment — Submitted filename: Response to Reviewers.docx [file pone.0251380.s002.docx]

Dear Editor,

We appreciate the significant contributions of the reviewers, which has given a face-lift to the manuscript. We are very grateful to them. However, our opinion differs with theirs on some minor issues, which could still be amended base on your recommendation. Details of the corrections are seen in the table below.

**Table of comments and corresponding action**

| **SN** | **Review Comments to the Author** | **Corresponding action** | **Explanation** |
| --- | --- | --- | --- |
| 1 | Including a map showing the study area would be helpful to the reader. | A map of the study area has been drawn and included in the manuscript. | We perfectly agree. |
| 2 | It would be useful for the authors to address the possibility of multicollinearity in the multiple regression analyses between income level and variables associated with income level (housing structure, window screens, etc). Was multicollinearity assessed, and is it something that needs to be addressed? This is the most important comment I have. | Multicollinearity analysis has been carried out and the results included in the manuscript. | We fully agree |
| 3 | Consider eliminating the p-values in tables 1 and 2 for the regression results. The 95% confidence intervals are the most important to include, and p-values are not as meaningful. | No action | Although the 95% confidence intervals are the most important, novices to statistics easily interpret p-values than confidence intervals. Does, having both will enable us to effectively communicate to a wider audience |
| 4 | I found the high infant prevalence to be interesting. The authors addressed the role of no built immunity as a reason for higher prevalence in this age group, which is an appropriate explanation. I am curious about the sleeping habits of infants in the study areas in relation to LLIN use- do infants typically sleep with a guardian? Is it known whether infants typically sleep under LLINs? | No action | There is no statistically significant difference (p=0.06) in LLINs usage between this age group and other age groups. Many other studies elsewhere have reported higher prevalence of malaria in this age group than the general population. |
| 5 | In figure 7, consider using the same type of figure to maintain consistency (either use all pie charts or all bar graphs) | No action | If we use all pie charts or all bar charts, the manuscript may become banal and too uniform to the viewer, to incite interest in reading onward. |
